# Factors associated with the CVD risk factors and body fat pattern of postmenopausal Hindu caste and Lodha tribal populations living in India: An exploratory study

**DOI:** 10.1186/s40695-023-00087-0

**Published:** 2023-04-25

**Authors:** Debasmita Kar, Subho Roy

**Affiliations:** Department of Anthropolgy, University of Calcutta, 35 Ballygunge Circular Road, 700019 Ballygunge, Kolkata, India

**Keywords:** Menopausal status, CVD, Blood glucose, Total cholesterol, Blood pressure

## Abstract

**Background:**

Loss in ovarian function during mid-life results in adverse changes in the cardiovascular profile of women. The association between CVD risk factors and menopause differ cross-culturally since several modifiable factors play significant roles in explaining CVD mortality in addition to differences in endogenous estrogen. Very few of the studies from the Indian subcontinent have been concerned with the menopause-specific CVD risk factors, particularly among the tribal groups. Thus, we intended to study the variations in body fat pattern and CVD risk factors between Hindu caste and Lodha tribal postmenopausal women and how these risk factors were associated with differential socio-economic, reproductive and menstrual characteristics and lifestyle variables. The Lodha tribal populations is considered a Particularly Vulnerable Group (PVTG) in this country.

**Methods:**

This cross-sectional study was conducted among the Bengali Hindu caste and Lodha tribal populations of the State of West Bengal, India covering three districts namely Howrah, Jhargram and East Midnapure. A total of 197 postmenopausal participants were recruited for this study (urban caste 69, rural caste 65 and rural Lodha 63). Data on blood glucose and total cholesterol levels, blood pressure, muscle mass, body fat distribution and sociodemographic, reproductive and menstrual history and lifestyle variables were collected following standard protocols. Analysis of variance (ANOVA) was applied to compare blood glucose, total cholesterol and blood pressure levels and body fat measures across the three populations. Stepwise multiple linear regression analysis was performed to find out the factors associated with CVD risk factors. The data were analyzed with the Statistical Package for Social Science version 20.0(IBM corporation, 2011).

**Results:**

This cross-sectional comparison of women at midlife, though exploratory in nature showed significant differences in body fat pattern and CVD risk factors between caste and tribal groups owing to socioeconomic disparities and, differences in reproductive characteristics and lifestyle factors.

**Conclusion:**

The caste and tribal populations differed significantly in body fat pattern and CVD risk factors and in the factors associated with CVD risk suggesting interplay between menopause and modifiable factors in explaining CVD risk factors during mid-life.

## Background

Cardiovascular disease (CVD) is recognized as the leading cause of death in both males and females, but the pathophysiological and clinical features of CVD are unique in the latter group. By far, CVD is the commonest cause of morbidity and mortality in postmenopausal women [1, 2]. Loss in ovarian function, and thereby a fall in the estrogen level during midlife results in adverse changes in glucose and insulin metabolism, body fat distribution, dyslipidemia, coagulation, fibrinolysis and vascular endothelial dysfunction, all of which increases the risk of CVD [3–10]. The Framingham heart study reported that women aged 50–59 years who experienced natural menopause are at four times more risk of CVD compared to the premenopausal women of same age range [11]. It is usually observed that women continue with a largely unchanged fat patterning from puberty to middle age. After menopause, excessive body fat starts to accumulate in the body due to the hormonal changes [3, 10]. During midlife, the fat patterning of the body changes from gynoid to android types leading to the development of central obesity among women [3, 10, 12]. Among Asian Indian women, CVD risk factors like central obesity are more prevalent among postmenopausal women compared to their premenopausal counterpart which later leads to the development of cardiovascular diseases [12].

Menopausal factors differ across cultural groups, so do the CVD risk factors. For example, the prevalence of CVD risk factors related to menopause varies across socioeconomic groups including risk factors such as early life events, family history, household stress, woman’s attitudes and behaviors towards menopause, age at attaining menopause and rural-urban residence [13–16]. In India, the occurrence of premature menopause (before age 40) is most common among rural agricultural workers, those who are non-literate, and those who have a low body mass index, signaling higher risks of CVD [17, 18]. In Indian subcontinent, studies addressing menopause focused primarily on the age of onset of menopause, attitude and perception towards menopause, severity of menopausal symptoms and its variations across socio-economic groups [19–22].

Very few of the studies from this subcontinent have been concerned with menopause specific characteristics that are related to CVD risk factors, particularly among the tribal groups [23–27]. Many of the ethnic minority groups of this country are socio-economically disadvantaged which exposes them to higher risks of inadequate food intake, poor hygiene and tobacco and alcohol consumption as well as lower access to health care [28, 29]. Thus, gaping disparities in health status of tribal women is observed when compared to metropolitan areas. This may in significant part occur from differing cultural practices and customs that in turn possibly results in the differing postmenopausal health outcomes among the caste and tribal women.

Studies reveal that the burden of CVD is now shifting from richer and better educated section of the population to the poor and less educated Sects. [34, 35]. In addition, the range of mean age at menopause of Indian women varies widely (41.9 and 49.4 years) leaving women of different cultural groups at increased risk of CVD [17, 30, 31]. Thus, an improved understanding of the concomitants associated with body fat pattern and increasing CVD risk factors during menopause among caste and tribal populations has become imperative. We hypothesized in this study that there will be a variation in body fat pattern and CVD risk factors between Bengali Hindu caste and Lodha tribal postmenopausal populations, within the state of West Bengal, India – a microcosm of ethnic, economic, rural/urban and health disparity. The intellectual merit of the present study is a perspective that incorporates multiple axes: menopause, ethnicity and urban/rural residence. In this cross-sectional study we intended to examine the variations in body fat pattern and CVD risk factors between Hindu caste and Lodha tribal postmenopausal women. The study further attempted to characterize the sociodemographic, reproductive and menstrual characteristics and lifestyle variables associated with these risk factors in postmenopausal women.

## Methods

### Study area

This cross-sectional study was conducted among the Bengali Hindu caste and Lodha tribal populations of the state of West Bengal, India. Lodha populations have been declared as one of the Particularly Vulnerable Tribal Groups (PVTGs) of this country on the basis of certain characteristics like low level of literacy, pre agricultural level of technology, and declining or stagnant population growth. Data for this study were collected from urban and rural areas of West Bengal, a state located in the eastern part of India. The study participants were selected from three districts of West Bengal namely- Howrah, East Midnapur and Jhargram. The urban Hindu caste (UHC) participants were selected from the Howrah district covering four municipal wards (41, 45, 46 and 48) of the Howrah Municipal Corporation (HMC) under the jurisdiction of Howrah *Sadar* Subdivision. The rural Hindu caste (RHC) participants were selected from Bhagawanpur 1 Community Development Block (CDB) of Egra sub-division of the district of East Midnapur. The rural Hindu caste participants have been selected from four villages of Bhagawanpur 1 CDB namely- Banlauri, Kalaberia, Kotlauri and Kuralbar. The rural Lodha (RL) participants were selected from Jhargram CDB of the Jhargram district. Eight Lodha villages namely- Sugnibasa, Shalukgeria, Ramchandrapur, Jorakhali, Suabasa, Kismat Bharatpur, Jaralata and Kanyaduba were selected for this study. The rural blocks were carefully selected considering the fact that these are far apart from urban facilities.

### Study participants

300 women were initially recruited for the present study on the basis of the criteria fixed for the study. The inclusion criteria were as follows: married with at least one surviving child, have attained natural menopause and have no reported history of metabolic disorders. A total of 103 women were excluded either because they did not meet the inclusion criteria (n = 55) or they did not agree (n = 48) to participate (25 from UHC, 30 from RHC and 48 from RL). Most of the refusals came from rural Lodha women (n = 48)since they were not comfortable in providing blood samples for estimating blood glucose and cholesterol levels. Women who went through surgical menopause (n = 28) or who took medicines (n = 27) for any metabolic abnormalities were excluded from the study. Unmarried women were excluded from the study to ensure that all the women had been exposed to certain reproductive events (pregnancy, use of contraception, lactation, parity). The hormonal changes during the above mentioned reproductive events are found to affect menopause as well as CVD [3, 10]. Finally, a total number of 197 postmenopausal participants were recruited for this study (69 UHC, 65 RHC and 63 RL). Postmenopausal status of the participants was defined as those who had stopped menstrual bleeding spontaneously for at least for the last twelve months [32]. The purpose of the research was explained and verbal and written informed consent was obtained from the participants. The study was approved by the Institutional Human Ethical committee, University of Calcutta.

### Data collection

The participants were interviewed in person by one of the authors (DK) to gather information about their sociodemographic and reproductive variables using a pretested structured schedule which was used in earlier studies conducted on the same population [30, 33]. Data on sociodemographic variables include age of the participants at the time of interview (years), educational levels and occupational types of the participants and their husbands and per capita monthly household expenditure (in Indian rupees). Data on menstrual characteristics include age at menarche (years) and menstrual bleeding length (days) prior to menopause. Reproductive history of the participants includes age at marriage (years), pregnancy record, contraceptive use, and breastfeeding history (in case of the last child) and age at menopause (years). Age at menarche was ascertained by asking the participants the actual date of the incident, if not then the nearest month. A few of the participants could recall their age at menarche in relation to a landmark and/or personal event (like her birthday) which occurred around the time of her menarche. Age at menopause was ascertained by asking the participants for how long their menstruation has stopped. A few of the participants recalled the time in relation to landmark events like her grandchild’s birthday or any other specific event that took place around the time of her last menstruation. Finally, age at menopause was calculated by subtracting the years after menopause from the age of the participants at the time of interview. Data on pregnancy record include number of pregnancies (number of live births, still births and miscarriages), and ages at first and last pregnancy.

Data on dietary behavior and food consumption pattern of the participants were taken using a pretested food frequency schedule [34]. The schedule includes eighteen food items that are commonly available within the study area and generally consumed by the participants. The participants were asked to report how often they consumed these food items within the past one week period prior to the date of interview. Each of the food items were categorized into eight responses, ranging from zero (representing never) to seven (all seven days in a week). Later, the food consumption pattern was categorized into three categories based on the following criteria- regularly (5–7 days consumption), occasionally (2–4 days consumption) and rarely/never(less than two days or never).

Data on physical activity were obtained using a pretested ‘physical activity’ schedule [35]. The schedule includes physical activity related to daily chores (cooking, washing dishes and clothes, mopping, dusting, watching television, listening music, walking, bicycling, physical exercise and doing crafts) of the participants. In addition to these daily chores, data were collected from RHC and RL populations about the duration of time spent by them on agricultural activities. For RL, in addition, information was collected on the duration of time they spent in collection of wood from the forest. The participants were asked to report how many minutes/day/week they engaged in these activities. Later, we estimated Metabolic Equivalent (MET) score following International Physical Activity Questionnaire (IPAQ). The physical activity level was represented as MET minutes a week. MET minutes represent the amount of energy spend in carrying out physical activity. The score assigned for particular activities were as follows- moderate physical activities (household chores) were considered as 4 METS, walking as 3.3 METS and vigorous physical activities (like, physical exercise, agriculture, and wood collection) as 8 METS. We calculated MET minutes/week by multiplying the given MET value with number of minutes the activity was carried out in a typical day and by the number of days the activity was undertaken in a typical week. For example, if a participant was engaged in mopping for at least 30 min (in a day) for 3 days in a week, then the MET score of the participant will be- 4 × 30 × 3 = 360 MET. Finally, we added the MET minutes estimated for each category of physical activity to get a total MET minutes of physical activity in a week. On this basis, we grouped the level of physical activity into three categories- High (up to 3000 MET minutes a week), moderate (up to 600 MET minutes a week)and low(below 600 MET minutes a week) level of activity [36, 37].

We measured random blood glucose (mg/dl) and total cholesterol levels (mg/dl) for all the participants. Although, there are certain limitations for using random blood glucose (RBG) test for estimation of diabetes, the International Diabetes Federation guidelines recommended screening for individuals with random glucose value ≥ 200 mg/dl [38, 39]. Blood samples were drawn from the tip of the second finger of the left hand and analyzed using blood glucose monitoring kit (Accu-check Active Blood glucose monitoring system, Model no. GB10803608) and Multi-care-in meter (Model no. IN2140129). Blood pressure (mmHg) of the participants was measured using Omron’s automatic blood pressure monitor (Model no. HEM-7121). Two consecutive readings of blood pressure were taken in the gap of ten minutes, and then the mean was calculated. Mean arterial pressure (MAP) (mmHg) for each participants was calculated using the formula- MAP = SBP + 2(DBP)/3.

Body weight (to the nearest of 0.1 kg), visceral fat, subcutaneous and skeletal fat for whole body and torso along with one anthropometric index, percent body fat (PBF) were measured for each participants in light clothing without shoes using Omron’s Body Composition Monitor(Model No. HBF-362). Muscle mass was measured by using Rossmax Body Fat Monitor (Model no. WF260). Each measurement was taken twice to assure the reliability of the instruments.

Anthropometric measurements like stature, waist circumference (WC) and hip circumference (HC) were measured following standard protocol [40]. Stature (to the nearest 0.1 cm) was measured using a portable GPM anthropometer for each participant standing without shoes on horizontal surface. WC and HC were measured to the nearest of 0.1 cm with a non-stretchable fiber glass insertion tape over light clothing. WC was measured at the minimum circumference of torso between the iliac crest and the rib cage. HC was measured horizontally at the level of maximum extension of the buttocks. Other anthropometric indices like waist-hip ratio(WHR), fat free mass(FFM) and fat mass(FM) were calculated following standard formulae:

WHR = WC (cm)/HC (cm)

FM = PBF/100× Weight (kg)

FFM = Weight- FM

The study was conducted during the time period of June 2018 to December 2019.

### Statistical analysis

We used descriptive statistics to find out the distribution of sociodemographic characteristics, reproductive and menstrual history, food consumption pattern and physical activities, anthropometric characteristics, body fat measures (subcutaneous and skeletal fat for whole body and torso, PBF, WC, HC, WHR, visceral fat, muscle mass, FM and FFM), blood glucose, total cholesterol and blood pressure levels of the participants. We applied Kolmogorov-Smirnov test to understand the distribution of each variable. Analysis of variance (ANOVA) was applied to compare blood glucose, total cholesterol and blood pressure levels and body fat measures across the three populations. Post-hoc test (Scheffe’s test) was applied to understand the differences between the study populations independently. Kruskal-Wallis test was applied as a substitute of ANOVA for those variables that did not follow the normal distribution. Two-tailed test of significance have been applied for the analysis. Multiple linear regression analysis was performed to find out the factors associated with body fat patterning and CVD risk factors. The variables of body fat patterning and CVD risk factors that differed significantly across the three study populations were selected as dependent variables in multiple linear regression analysis. Sociodemographic, reproductive and menstrual history and lifestyle variables (food habit and physical activities) were selected as independent variables in the model. Stepwise multiple linear regression was conducted to find out the best fitted model. Collinearity of the independent variables was also checked. The WHO (2008) Asian specific cutoff for women has been used to determine central obesity- for WC > 88 cm was considered as risk category, while ≥ 0.85 was considered as risk category in case of WHR. Participants with blood sugar level < 200 mg/dl were considered as non- diabetic and those with ≥ 200 mg/dl as diabetic; participants with total cholesterol level ≥ 240 mg/dl were labeled as high cholesterol level and those, with < 240 mg/dl were labeled as normal; participants with SBP ≥ 140 mmHg and DBP ≥ 90 mmHg and Mean Arterial Pressure ≥ 100 were considered to be hypertensive [41]. Chi square tests were performed to understand the distribution of these CVD risk factors across the three populations. A minimum ‘p’ value of 0.05 was considered as statistically significant level for all inferential statistics. The data were analysed with the help of statistical package for social science version 20.0(IBM corporation, 2011).

## Results

Age of the participants reported at the time of interview, working status, occupational and educational categories and per capita monthly household expenditure of the participants differed significantly across the three populations. The majority of the participants from RHC (71.8%) and RL (80.5%) were engaged in agricultural activities. The majority of the participants from RL (95.2%) were non-literate (Table 1).

**Table 1:** Sociodemographic characteristics of the study participants by Urban Hindu caste, Rural Hindu casteand Rural Lodha tribe, West Bengal India (n = 197)

Sociodemographic variables	Urban Hindu caste (UHC) (n = 69)	Rural Hindu caste (RHC) (n = 65)	Rural Lodha (RL) (n = 63)	F value/Kruskal-wallis test/ χ2 test	p value
Mean age at the time of interview(mean ± sd)	53.12 ± 7.48	55.45 ± 8.17	59.35 ± 6.20	12.02	0.0001
Working status					
Working (n (%)) Non-working (n (%))	22 (31.9) 47 (68.1)	39 (60.0) 26 (40.0)	41 (65.1) 22 (34.9)	17.16	0.0001
Service (n (%) ) Business (n (%) ) Agriculture and wood collection (n (%))	2 (9.1) 20 (90.2) -	- 11 (28.2) 28 (71.8)	- 8 (19.5) 33 (80.5)	45.05	0.0001
Educational category of the participants					
Literate (n (%) ) Non-literate (n (%) )	62 (89.9) 7 (10.1)	60 (92.3) 5 (7.7)	3 (4.8) 60 (95.2)	137.65	0.0001
Educational category of the spouses					
Literate (n (%) ) Non-literate (n (%) )	69 (100) -	58 (89.2) 7 (10.8)	6 (9.5) 57 (90.5)	158.10	0.0001
Per capita monthly household expenditure (INR)	151.51*	79.80*	59.44*	97.35	0.0001

*mean rank; figures in the parentheses indicate percentage values

The reproductive and menstrual characteristics of the participants differed significantly across the three populations. None of the participants from RL had used any kind of contraceptives. Mean age at menopause was found to be significantly earlier among the UHC followed by the RHC and RL (Table 2).

**Table 2:** Menstrual and reproductive history of the participants by Urban Hindu caste, Rural Hindu caste and Rural Lodha tribe, West ,Bengal India(n = 197)

Menstrual and reproductive characteristics	Urban Hindu Caste (n = 69)	Rural Hindu Caste (n = 65)	Rural Lodha (n = 63)	F value/Kruskal-wallis test/ χ2 test	p value
Mean age (years) at menarche(mean ± sd)	12.41 ± 1.85	14.18 ± 1.73	13.79 ± 0.98	23.45	0.0001
Mean age (years) at marriage(mean ± sd)	19.25 ± 4.77	16.29 ± 2.49	14.22 ± 5.56	21.18	0.0001
Age at first pregnancy (mean ± sd)	21.22 ± 4.99	18.26 ± 2.34	17.56 ± 2.57	19.95	0.0001
Age at last pregnancy (mean ± sd)	26.26 ± 5.28	30.18 ± 5.52	26.56 ± 5.97	10.01	0.0001
Number of pregnancies (n (%))					
One Two More than two	11 (15.9) 21 (30.4) 37 (53.6)	1 (1.5) 6 (9.2) 58 (89.2)	5 (7.9) 12 (19.0) 46 (73.0)	21.68	0.0001
Ever experience of fetal loss (n (%))					
Yes No	32 (46.4) 37 (53.6)	27(41.5) 38(58.5)	9 (14.3) 54 (85.7)	17.12	0.0001
Mean duration of breastfeeding(months)	75.14*	113.21	110.48	19.09	0.0001
Ever use of contraceptive (n (%))					
Yes No	21 (30.4) 48 (69.6)	23 (35.4) 42 (64.6)	- 63(100)	27.10	0.0001
Mean age (years) at menopause(mean ± sd)	42.68 ± 7.80	46.56 ± 4.78	48.74 ± 5.18	16.51	0.0001

*mean rank; figures in the parentheses indicate percentage values

Participants from the three groups differed significantly in their consumption patterns. Barring regular consumption of roots and tubers, soya and meat, the frequency of regular consumption of other food items seems to be significantly higher among the UHC and RHC participants compared to RL. The physical activity level and the frequency of substance abuse differed significantly across the three groups. The majority of the RL participants (84.1%) were engaged in high level of physical activity compared to UHC and RHC. The majority of the participants from RL (90.5%) reported chewing tobacco on a regular basis followed by UHC and RHC. The majority of the participants from RL (79.4%) reported consuming alcohol on a regular basis, while none of the participants from the caste groups reported consuming alcohol (Table 3).

**Table 3:** Pattern of food consumption and physical activity level among the participants by Urban Hindu caste, Rural Hindu caste and Rural Lodha tribe, West ,Bengal India(n = 197) (last seven days recall period)

Food items	Urban Hindu Caste (n = 69) N (%)	Rural Hindu Caste (n = 65) N (%)	Rural Lodha (n = 63) N (%)	Chi square test/ Fisher’s exact test	p value
Intake of legumes, beans, seeds Regularly Occasionally Rarely/never	39 (56.5) 8 (11.6) 22 (31.9)	30 (46.2) 1 (1.5) 34 (52.3)	18 (28.6) 16 (25.4) 29 (46.0)	24.80	0.0001
Intake of green vegetables Regularly Occasionally Rarely/never	63 (91.3) 2 (2.9) 4 (5.8)	61 (93.8) - 4 (6.2)	31 (49.2) 16 (25.4) 16 (25.4)	46.96	0.0001
Intake of roots and tubers Regularly Occasionally Rarely/never	54 (78.3) - 15 (15.7)	47 (72.3) - 18 (27.7)	52 (82.5) 3 (4.8) 8 (12.7)	8.25	0.04
Intake of soya products Regularly Occasionally Rarely/never	4 (5.8) 7 (10.1) 58 (84.1)	4 (6.2) 2 (3.1) 59 (90.8)	12 (19.0) 15 (23.8) 36 (57.1)	22.93	0.0001
Intake of meat Regularly Occasionally Rarely/never	- 5 (7.2) 64 (92.8)	1 (1.5) 6 (9.2) 58 (89.2)	17 (27.0) 31 (33.3) 25 (39.7)	60.57	0.0001
Intake of fish Regularly Occasionally Rarely/never	50 (72.5) 7 (10.1) 12 (17.4)	56 (86.2) 5 (7.7) 4 (6.2)	11 (17.5) 27 (42.9) 25 (39.7)	73.61	0.0001
Intake of egg Regularly Occasionally Rarely/never	17 (24.6) 6 (8.7) 46 (66.7)	11 (16.9) 11 (16.9) 43 (66.2)	4 (6.3) 22 (34.9) 37 (58.7)	18.81	0.001
Intake of fruits Regularly Occasionally Rarely/never	22 (31.9) 8 (11.6) 39 (56.6)	9 (13.8) - 56 (86.2)	1 (1.6) 6 (9.5) 56 (88.9)	35.20	0.0001
Intake of milk Regularly Occasionally Rarely/never	20 (29.0) 1 (1.4) 48 (69.6)	13 (20.0) - 52 (80.0)	3 (4.8) - 60 (95.2)	16.38	0.001
Intake of snacks Regularly Occasionally Rarely/never	60 (87.0) 1 (1.4) 8 (11.6)	40 (61.5) - 25 (38.5)	31 (49.2) 5 (7.9) 27 (42.9)	27.46	0.0001
Intake of sweets Regularly Occasionally Rarely/never	38 (55.1) 4 (5.8) 27 (39.1)	20 (30.8) 1 (1.5) 44 (67.7)	- 5 (7.9) 58 (92.1)	62.53	0.0001
Intake of tea Regularly Occasionally Rarely/never	61 (88.4) - 8 (11.6)	31 (47.7) - 34 (52.3)	49 (77.8) - 14 (22.2)	28.36	0.0001
Physical activity High (n (%)) Moderate Low	55 (79.7) 13 (18.8) 1 (1.4)	46 (70.8) 11 (16.9) 8 (12.3)	53 (84.1) 8 (12.7) 2 (3.2)	9.58	0.04
Habit of tobacco chewing (n (%)) Yes No	11(15.9) 58 (84.1)	6 (9.2) 59 (90.8)	57(90.5) 6 (9.5)	111.20	0.0001
Alcohol consumption (n (%) ) Yes No	- 69 (100)	- 65 (100)	50 (79.4) 13 (20.6)	-	-

**Table 4:** Distributions of fat patterning and CVD risk factors among urban Hindu caste, rural Hindu caste d rural Lodha tribal group, West Bengal, India (n = 197)

Variables	Urban Hindu Caste (n = 69)	Rural Hindu Caste (n = 65)	Rural Lodha (n = 63)	Fvalue/ Kruskal-Wallis test	p value	Post-hoc test
	mean ± sd	mean ± sd	mean ± sd			
Body mass index(BMI)	25.21 ± 4.23	21.12 ± 3.65	17.93 ± 3.36	61.59	0.0001	UHC vs. RHC = 0.001 UHC vs. RL = 0.001 RHC vs. RL = 0.001
WHR	0.95 ± 0.08	0.88 ± 0.09	0.84 ± 0.08	23.40	0.0001	UHC vs. RHC = 0.0001 UHC vs. RL = 0.0001 RHC vs. RL = 0.03
Visceral fat	8.86 ± 4.94	5.16 ± 3.05	2.38 ± 1.87	54.64	0.0001	UHC vs. RHC = 0.0001 UHC vs. RL = 0.0001 RHC vs. RL = 0.0001
Whole body subcutaneous fat percentage	31.01 ± 4.70	27.27 ± 4.48	23.36 ± 3.56	52.17	0.0001	UHC vs. RHC = 0.0001 UHC vs. RL = 0.0001 RHC vs. RL = 0.0001
Whole body skeletal fat percentage	21.69 ± 1.79	21.57 ± 2.60	22.13 ± 2.37	1.06	0.34	UHC vs. RHC = 0.95 UHC vs. RL = 0.54 RHC vs. RL = 0.38
Trunk subcutaneous fat percentage	27.90 ± 4.57	24.81 ± 4.69	21.44 ± 4.01	34.72	0.0001	UHC vs. RHC = 0.0001 UHC vs. RL = 0.0001 RHC vs. RL = 0.0001
Trunk skeletal fat percentage	16.03 ± 1.91	16.48 ± 2.65	17.40 ± 2.21	6.095	0.003	UHC vs. RHC = 0.52 UHC vs. RL = 0.003 RHC vs. RL = 0.07
Arm subcutaneous fat percentage	48.40 ± 5.28	46.34 ± 5.90	42.56 ± 6.34	16.68	0.0001	UHC vs. RHC = 0.12 UHC vs. RL = 0.0001 RHC vs. RL = 0.0002
Arm skeletal fat percentage	23.60 ± 3.87	25.33 ± 3.38	27.40 ± 2.79	20.56	0.0001	UHC vs. RHC = 0.01 UHC vs. RL = 0.0001 RHC vs. RL = 0.003
Leg subcutaneous fat percentage	42.10 ± 5.42	36.63 ± 7.10	31.95 ± 4.80	49.62	0.0001	UHC vs. RHC = 0.0001 UHC vs. RL = 0.0001 RHC vs. RL = 0.0001
Leg skeletal fat	34.28 ± 2.47	32.42 ± 3.40	30.97 ± 3.02	20.50	0.0001	UHC vs. RHC = 0.002 UHC vs. RL = 0.0001 RHC vs. RL = 0.02
Blood glucose level(mg/dl	107.14*	114.85	73.73	18.81	0.0001	UHC vs. RHC = 0.49 UHC vs. RL = 0.001 RHC vs. RL = 0.0001
SBP(mmHg)	136.49 ± 21.48	135.37 ± 22.96	152.65 ± 28.77	9.99	0.0001	UHC vs. RHC = 0.96 UHC vs. RL = 0.001 RHC vs. RL = 0.0001
DBP(mmHg)	87.14 ± 10.72	85.83 ± 10.60	95.44 ± 15.14	11.54	0.0001	UHC vs. RHC = 0.82 UHC vs. RL = 0.001 RHC vs. RL = 0.0001
Total cholesterol (mg/dl)	110.35*	85.43	100.57	6.46	0.03	UHC vs. RHC = 0.008 UHC vs. RL = 0.39 RHC vs. RL = 0.17
FM	21.60 ± 5.79	16.73 ± 4.80	12.82 ± 3.59	54.44	0.0001	UHC vs. RHC = 0.0001 UHC vs. RL = 0.0001 RHC vs. RL = 0.0001
FFM	35.88 ± 5.46	30.44 ± 6.40	26.22 ± 4.76	49.66	0.0001	UHC vs. RHC = 0.0001 UHC vs. RL = 0.0001 RHC vs. RL = 0.0001
Muscle mass	28.84 ± 3.28	30.82 ± 3.28	33.26 ± 2.67	33.44	0.0001	UHC vs. RHC = 0.001 UHC vs. RL = 0.0001 RHC vs. RL = 0.0001
MAP	89.16*	84.95	124.27	18.38	0.0001	UHC vs. RHC = 0.62 UHC vs. RL = 0.0001 RHC vs. RL = 0.0001

*mean rank

**Table 5:** Factors associated with fat patterning and CVD risk factors: Multiple linear regression

Dependent variable	Independent variables	Unstandardized coefficients	t value	p value	CI at 95%		R square
					lower	upper	
BMI	Urban residence Completed years of education of the spouses Total number of pregnancies	3.98 0.35 0.37	5.31 4.81 2.30	0.0001 0.0001 0.02	2.50 0.21 0.05	5.45 0.50 0.70	0.40
PBF	Completed years of education of the spouses Age of the participants at the time of interview Urban residence Age at first pregnancy Tobacco chewing	0.29 0.29 2.76 -0.22 -1.75	2.81 6.09 3.06 -2.30 -2.16	0.005 0.0001 0.003 0.02 0.03	0.08 0.19 0.98 -0.41 -3.35	0.50 0.38 4.54 -0.03 -0.15	0.27
WHR	Urban residence Exclusively homemaker	0.07 0.03	5.24 2.94	0.0001 0.004	0.04 0.01	0.10 0.06	0.20
Visceral fat	Urban residence Completed years of education of the spouses Age of the participants at the time of interview Fish consumption	3.74 0.25 0.10 0.27	5.48 3.50 2.91 2.68	0.0001 0.001 0.004 0.008	2.39 0.11 0.03 0.07	5.09 0.39 0.17 0.47	0.39
Whole body subcutaneous fat	Completed years of education of the spouses Urban residence Age of the participants at the time of interview Tobacco chewing	0.32 3.71 0.16 -2.06	3.56 4.58 3.87 -2.82	0.0001 0.0001 0.0001 0.005	0.14 2.11 0.08 -3.51	0.51 5.29 0.25 -0.62	0.40
Trunk subcutaneous fat	Completed years of education of the spouses Age of the participant’s at the time of interview Urban residence Tobacco chewing Age at first pregnancy	0.34 0.21 3.54 -1.91 -0.18	3.55 4.87 4.30 -2.58 -2.04	0.0001 0.0001 0.0001 0.01 0.04	0.15 0.12 1.91 -3.36 -0.35	0.53 0.29 5.16 -0.45 -0.007	0.35
Trunk skeletal fat	Age of the participants at the time of interview Urban residence Tobacco chewing Exclusively homemaker Age at first pregnancy	-0.17 -1.34 1.21 -0.83 0.10	-8.77 -4.11 4.18 -2.91 2.69	0.0001 0.0001 0.0001 0.004 0.008	-0.21 -1.99 0.64 -1.40 0.02	-0.13 -0.07 1.78 -0.27 0.17	0.41
Arm subcutaneous fat	Completed years of education of the spouses Age of the participants at the time of interview Age at marriage Urban residence Tobacco chewing	0.44 0.23 -0.22 2.43 -2.01	3.50 3.96 -2.44 2.23 -2.05	0.001 0.0001 0.01 0.02 0.04	0.19 0.11 -0.41 0.28 -3.95	0.70 0.34 -0.04 4.58 -0.07	0.24
Arm skeletal fat	Completed years of education of the spouses Age of the participants at the time of interview Urban residence Age at first pregnancy Fish consumption Milk consumption	-0.31 -0.18 -2.18 0.15 -0.21 0.18	-4.66 -5.84 -3.60 2.41 -2.42 2.09	0.0001 0.0001 0.0001 0.01 0.01 0.03	-0.45 -0.24 -3.37 0.02 -0.39 0.01	-0.18 -0.12 -0.98 0.28 -0.04 0.35	0.34
Leg subcutaneous fat	Completed years of education of the spouses Urban residence Fish consumption	0.42 4.85 0.42	3.59 4.34 2.53	0.0001 0.0001 0.01	0.19 2.65 0.09	0.65 7.06 0.75	0.36
Leg skeletal fat	Age of the participants at the time of interview Total number of wastage Age at first pregnancy Urban residence Age at menopause	-0.21 0.88 0.15 1.32 0.08	-7.14 4.14 2.95 3.01 2.59	0.0001 0.0001 0.004 0.003 0.01	-0.27 0.46 0.05 0.45 0.02	-0.15 1.30 0.25 2.19 0.15	0.41
Blood glucose level	Age at menopause Physical activity level Meat consumption	-1.75 -0.002 -3.34	-3.50 -2.50 -2.01	0.001 0.01 0.04	-2.74 -0.004 -6.61	-0.76 -0.001 -0.06	0.12
Blood total cholesterol level	Age at marriage Urban residence	-2.19 19.48	-2.80 2.43	0.006 0.01	-3.73 3.68	-0.64 35.29	0.05
Muscle mass	Urban residence Age of the participants at the time of interview Completed years of education of the spouses Fish consumption Milk consumption Total number of wastage	-2.60 -0.22 -0.23 -0.23 0.20 -0.55	-4.94 -7.80 -4.10 -2.93 2.72 -2.35	0.0001 0.0001 0.0001 0.004 0.007 0.01	-3.64 -0.27 -0.35 -0.38 0.05 -1.01	-1.56 -0.16 -0.12 -0.07 0.35 -0.09	0.44
Fat mass	Completed years of education of the spouses Urban residence Exclusively homemaker Fish consumption Milk consumption	0.45 3.56 1.56 0.33 -0.26	4.74 3.93 2.25 2.45 -2.04	0.0001 0.0001 0.02 0.01 0.04	0.26 1.77 0.19 0.06 -0.52	0.64 5.35 2.93 0.59 -0.009	0.42
Mean arterial pressure	Age of the participants at the time of interview Tobacco chewing	0.59 6.57	4.02 2.95	0.0001 0.004	0.30 2.18	0.87 10.95	0.14

All variables measuring fat patterning (except whole body skeletal fat) and CVD risk factors (SBP, DBP, MAP, random glucose, total cholesterol) differed significantly across the three populations (Table 4). The table also shows that expect for skeletal fat related to trunk and arm, muscle mass, SBP, DBP and MAP, the UHC had the highest values, followed by RHC and RL. The post hoc tests also suggested significant differences between UHC and RHC and RL and UHC and RL (Table 4).

Results of the multiple linear regressions are provided in Table 5. All of the body fat measures (barring WHR, trunk and skeletal fat) showed positive associations with the spouses completed years of education, while muscle mass showed a negative association. All of the body fat measures and total cholesterol level showed positive association with urban residential status. While skeletal fat related to trunk and arm, and muscle mass showed negative associations. PBF, visceral fat, subcutaneous fat related to whole body, trunk and arm and MAP increased with increasing in age of the participants; torso skeletal fat and muscle mass were likely to decrease with age of the participants. Blood glucose level was negatively associated with age at menopause, meat consumption and physical activity level. Participants, who were exclusively homemakers showed a positive association with WHR and FM. PBF, subcutaneous fat related to whole body and trunk showed negative association with tobacco chewing, while MAP and skeletal fat related to trunk showed positive association. PBF and trunk subcutaneous fat were negatively associated with age at first pregnancy. The R square values indicate that the models can explain 12–60% of the variance (Table 5).

**Table 6:** Distribution of CVD risk factors among Urban Hindu caste, Rural Hindu caste and Rural Lodha tribal group, West Bengal India (n = 197)

CVD risk factors	Urban Hindu Caste (n = 69)	Rural Hindu Caste (n = 65)	Rural Lodha (n = 63)	χ2 test	p value
Blood glucose level(mg/dl)					
Non-diabetic(< 200) Diabetic (≥ 200)	62 (89.9) 7 (10.1)	63 (96.9) 2 (3.1)	62 (98.4) 1 (1.6)	4.94	0.08
Waist circumference					
Normal (< 88) Risk (> 88)	30 (43.5) 39 (56.5)	56 (86.2) 9 (13.8)	62 (98.4) 1 (1.6)	61.51	0.0001
WHR					
Normal (< 0.85) Risk (≥ 0.85)	12 (17.4) 57 (82.6)	21 (32.3) 44 (67.7)	40 (63.5) 23 (36.5)	30.94	0.0001
SBP(mmHg)					
Normal (≤ 139) Hypertensive (≥ 140)	43 (62.3) 36 (37.7)	40 (61.5) 25 (38.5)	21 (33.3) 42 (66.7)	14.08	0.001
DBP(mmHg)					
Normal (≤ 89) Hypertensive (≥ 90)	45 (65.2) 24 (34.8)	44 (67.7) 21 (32.3)	23 (36.5) 40 (63.5)	15.71	0.0001
MAP					
Normal(< 100) Hypertensive(≥ 100)	31 (44.9) 38 (55.1)	29 (44.6) 36 (55.4)	14 (22.2) 49 (77.8)	9.29	0.01
Total cholesterol level					
Normal(< 240) High(≥ 240)	59 (85.5) 10 (14.5)	60 (92.3) 5 (7.7)	51 (81.0) 12 (19.0)	3.54	0.17

*(WHO, 2008)

Central obesity was higher among the UHC and RHC participants. 77.8% of the RL participants were found to be hypertensive, followed by RHC (55.4%) and UHC participants (55.1%). The incidence of diabetes and high total cholesterol level was found to be lower among all the populations (Table 6).

## Discussion

Our study reveals that UHC participants have higher body fat distribution and are more prone to CVD risk factors (central obesity, blood glucose level, total cholesterol level) compared to the RHC and RL participants. Urbanization and economic development may bring improvement in the socio-economic status (SES) and push a population to nutritional transition and to adopt sedentary lifestyle [42, 43]. This makeover of lifestyle leads to the increased prevalence in central obesity, glycemic abnormality, dyslipidemia and hypertension irrespective of their place of residence [44, 45]. A number of previous studies show consistency with our study where urban women showed higher body fat distribution compared to their rural counterpart [46, 47]. For example, studies from India and western countries show positive association between education, income and body fat distribution [48, 49], but the trend is not universal [50–52].

Completed years of education of the spouses of the participants shows positive association with all the body fat measures in our study; this is in disagreement with [53, 54] or non-significant in some earlier studies [55, 56]. For example, a study conducted among Japanese women showed educational attainment of the spouses of the participants at high school level or lower had higher risk of obesity compared with women whose spouses attained higher level of education than the former group [54]. In our study the literacy rate of the spouses of the participants showed positive association with central obesity. Perhaps, attainment of higher educational level of the spouses is associated with the higher social position and higher income leading to over nutrition and adoption of sedentary lifestyle. The prevalence of central obesity of rural and urban caste participants was 82.6% and 67.7% respectively consistent with some Indian studies [57, 58].

We found age at menopause to be a significant predictor for higher blood glucose level. Early cessation of menstruation is associated with suppression of estrogen at a premature age. This phenomenon affects insulin resistance by lowering the sex hormone binding globulin concentration of the blood and changing the body fat distribution from gynoid to android type [59, 60]. For example, in one US based study, women who experienced natural menopause before 40 years had a 50% higher chance of CVD related mortality than those reporting menopause at 50 years or later [61]. The UHC participants of our study reached menopause at an earlier age compared to the RHC and RL participants (48.74 ± 5.18). So, the early age at menopause might be a reason for the increased blood glucose level among the urban Hindu caste populations.

Our study shows that the RL participants are more prone to hypertension and have higher total cholesterol level compared to the UHC and RHC participants, but the trend is reverse for blood glucose level and in the prevalence of central obesity. We found an association between chewing of tobacco and increase in mean arterial pressure and decrease in body fat (PBF, subcutaneous fat related to whole body and trunk). A study conducted in an Indian city (Mumbai) showed that all forms of tobacco use were associated with low body fat, irrespective of age, education, and religion [62]. In our study, the majority of the RL participants chew tobacco on a regular basis which might be a reason behind their low body fat and increased hypertension because tobacco produce free radicals that deplete antioxidants like Vitamins C, E, and carotenoids and cause oxidative damage to DNA, proteins and lipids [63, 64]. Studies show that consumption of anti-oxidant-rich foods such as green-leafy vegetables and fruits perhaps reduce the oxidative stress caused by tobacco; but the Lodha participants of our study lack these items in their dietary practices and thereby remains at a risk of tobacco induced oxidative stress.

Our study found physical activity to have an inverse association with the blood glucose level; this could be a reason behind the lower incidence of diabetes among the tribal participants. A recent study conducted among Chinese women reveals that a higher degree of physical activity was associated with lower blood glucose level regardless of sex, menopausal status and first-degree family history of diabetes [65]. Physical inactivity and obesity are critical and modifiable risk factors of diabetes; this could be a justification of the increased blood glucose level among the urban Hindu caste participants as most of them were found to be engaged in sedentary activities.

We observed an inverse association between consumption of meat and blood glucose level. Studies show that regular intake of meat leads to increased blood glucose level due to iron overload [66, 67], which is contradictory to our findings. The majority of the RL participants consume meat (poultry product) on a regular basis. Poultry products contains less calories, highly digestible (with low levels of collagen) proteins, unsaturated lipids, B group vitamins and minerals like iron, zinc and copper and also has a low glycemic index value which helps in controlling the blood glucose level [68]. Future studies are required to confirm or refute this hypothesis.

Our study further revealed that working status of the participants was significantly associated with body fat measures like waist-hip ratio and fat mass showing consistency with some previous studies [69–71]. For example,a study conducted in an Indian city (Mumbai) reported that working women have lower body fat distribution compared to the non-working women [71]. The UHC participants of our study are mostly non-working, while the majority of the participants from RHC and RL are engaged in high intensity physical activity like agriculture and wood collection. This could be a reason behind the increased level of body fat among the urban participants compared to their rural counterparts.

Pregnancy and childbirth can additionally modify a woman’s risk of midlife obesity and CVD [72–74]. Women’s reproductive history may influence short and long term cardio-metabolic and cardiovascular trajectories later in life. Reproductive characteristics and pregnancy history in women are increasingly recognized in cardiovascular and obstetric society guidelines, with premature age of menopause and adverse pregnancy outcomes in particular now codified as risk-enhancing factors for CVD [75]. Our study showed that parity is positively associated with BMI, while age at first pregnancy had an inverse association with PBF and subcutaneous fat of trunk and a positive association with the skeletal fat of arms and legs. Childbirth at a younger age and increased parity have been independently associated with central obesity for women later in life due to increased stress or changes in the lifestyle factors which is in partial agreement with our study [72, 76]. For example, studies on Korean and US postmenopausal women showed similar results [73, 74]. Younger age at first childbirth is sometimes associated with disruption in education and occupational attainment which can increase the risk of obesity. Earlier exposure to high levels of estrogen by early pregnancy may lead to an increased body fat which in turn is speculated to influence CVD risk factors through complex interaction between oxidative stress, inflammation, the renin-angiotensin-aldosterone system, and the renal sympathetic nervous system [73, 74]. During pregnancy, the release of corticotrophin releasing hormone from the placenta drives the hypothalamic pituitary-adrenal axis and cortisol concentrations in pregnant women and may contribute to the pathophysiological mechanism of obesity later in life [78]. But, a recent study conducted among Korean postmenopausal women showed no significant association between age at first childbirth and central obesity after controlling for confounding variables [77]. We found an association between higher incidence of central obesity and higher parity among rural caste participants. Carrying a child to full term is associated with maternal metabolic changes and weight gain that persist after pregnancy. This could partially explain why RHC participants have higher incidence of central obesity. The RL participants of our study mostly conceived for the first time at a younger age (below 19 years) and show higher parity compared to the other two groups. But the prevalence of central obesity is not predominant among the Lodhas perhaps due to their high intensity physical activity.

Age of the participants showed significant positive association with body fat and mean arterial pressure showing consistency with some previous studies where degenerative effects of age have been reported [79, 80]. This could be attributed to the age specific changes at the cellular level, including oxidative stress, inflammation, and apoptosis, changing in the calcium plumping capacity, and overall myocardial deterioration and degeneration [81].

This exploratory study confirms to our hypothesis that significant differences exist in body fat pattern and CVD risk factors between caste and tribal groups of postmenopausal women owing to the socioeconomic disparities, differences in reproductive characteristic and lifestyle factors.

There are certain limitations in this study. Estimation of fasting blood glucose level and the total lipid profile analysis, taking a larger sample size and a closer observation on the lifestyle practices on the participants could have improved the scientific rigor of the study. Postmenopausal women become susceptible to health problems by reason of genetics, differences in attitude and perception and finally, their access to adequate health care services. Thus, inclusion of the data on these domains would give a better understanding of the cross-cultural difference in CVD risk factors of the caste and tribal group of the present study.

## Conclusions

In the present study, all the three groups of postmenopausal women (UHC, RHC and RL) differed significantly in body fat distribution and CVD risk factors, reinforcing the role of both modifiable factors and endogenous estrogen in the occurrence of CVD. Hence, research on cross cultural differences in postmenopausal health outcomes is needed for better understanding of menopause specific CVD risk factors. This will help policy makers to develop appropriate measures for midlife women and will present an opportunity for prevention and early intervention.

The authors are thankful to the University Grants Commission for the UGC fellowship under which the fieldwork was conducted.

## Data Availability

The datasets used/or analyzed during the current study are available from the corresponding author on reasonable request.

## Abbreviations

CVD: Cardiovascular Disease
BMI: Body Mass Index
PBF: Percent Body Fat
WC: Waist circumference
HC: Hip circumference
WHR: Waist Hip ratio
FFM: Fat Free Mass
FM: Fat Mass
SBP: Systolic Blood Pressure
DBP: Diastolic Blood Pressure
MAP: Mean Arterial Pressure
PVTGs: Particularly Vulnerable Tribal groups
UHC: Urban Hindu Caste
RHC: Rural Hindu Caste
RL: Rural Lodha
